# Fulminant Type 1 Diabetes Mellitus Associated With Drug Hypersensitivity and Epstein–Barr Virus Infection: A Case Report

**DOI:** 10.3389/fphar.2022.884878

**Published:** 2022-07-08

**Authors:** Xing-Yu Chen, Cong Wang, Shizhi Chen, Mingyuan Tian, Xin Wang, Lili Zhang

**Affiliations:** ^1^ Department of Endocrinology, The Second Affiliated Hospital, Chongqing Medical University, Chongqing, China; ^2^ Department of Clinical Laboratory, The Second Affiliated Hospital, Chongqing Medical University, Chongqing, China

**Keywords:** fulminant type 1 diabetes mellitus, Epstain-Barr virus, drug hypersensitivity, gene, case report

## Abstract

**Background:** Fulminant type 1 diabetes mellitus (FT1DM) is a new subtype of type 1 diabetes, first proposed by Japanese scholars in 2000. Herein, the functions of the islets are rapidly destroyed. Its pathogenesis is related to viral infection. Most people have been infected with Epstein–Barr virus (EBV), and many people have also suffered from drug hypersensitivity, however, few cases of FT1DM which were caused by both of the two conditions have been reported. Thus, below, we describe one such valuable case.

**Case Summary:** The plasma glucose levels of a 73‐year‐old man diagnosed with drug-induced dermatitis showed a sudden increase (42 mmol/L) during methylprednisolone therapy. The urine ketone test was positive. The glycated hemoglobin level was 7%, endogenous insulin secretion decreased significantly, and the islet-related autoantibodies were negative. The patient was diagnosed with FT1DM. The lymphocyte EBV-DNA showed high copies numbers. The general condition of the patient improved after symptomatic treatment with insulin. However, the systemic allergic reaction aggravated after the use of iodinated contrast agents, prednisone, and thymic pentapeptide. The re-test for EBV-DNA showed significantly high relative levels, thus indicating the presence of EBV infection. We think that drug hypersensitivity and EBV infection together led to FT1DM in this case. After an indication for multiple daily insulin therapy, the patient’s blood glucose was quickly controlled and he was discharged on the 38th-day post-admission.

**Conclusion:** FT1DM is a rare case, however, drug hypersensitivity and EBV infection are not rare in the population. This is a rare case of FT1DM caused by drug hypersensitivity reaction and EBV infection. Through this case report, we emphasize the importance of the relationship between drug hypersensitivity, EBV infection and FT1DM and vigilance for the occurrence of FT1DM among hypersensitive individuals in clinical practice.

## Introduction

Type 1 diabetes mellitus (T1DM), classified into the autoimmune and idiopathic types, is characterized by insufficient insulin production due to the destruction of pancreatic β‐cells. Fulminant type 1 diabetes mellitus (FT1DM) is a subtype of idiopathic T1DM, wherein a large number of pancreatic β‐cells are destroyed in a short time; its progression is rapid. The disease is characterized by the onset of diabetic ketosis or ketoacidosis within a few days of the onset of symptoms of hyperglycemia. FT1DM accounts for approximately 20% of the acute onset T1DM in Japan, and 7% of the all T1DM in Korea (Imagawa et al., 2003; Cho et al., 2007).

Recent studies show that the pathogenesis of FT1DM is associated with viral infections, including enterovirus, human herpesvirus 6, or cytomegalovirus (Imagawa and Hanafusa, 2011). The viruses in the pancreas trigger the relevant immune responses, and these activated immune cells cause damage to pancreatic β‐cells (Hosokawa et al., 2019). Immune-related FT1DM has also been reported following drug-induced hypersensitivity syndrome (DIHS). FT1DM is rarely encountered in clinical practice, and the cases of this disease caused by drug hypersensitivity and EBV infection are scarcely reported. We recently diagnosed a patient with FT1DM. The sudden sharp decline in pancreatic β‐cell functions was attributed to drug hypersensitivity and EBV infection.

## Case Presentation

**FIGURE 1:**
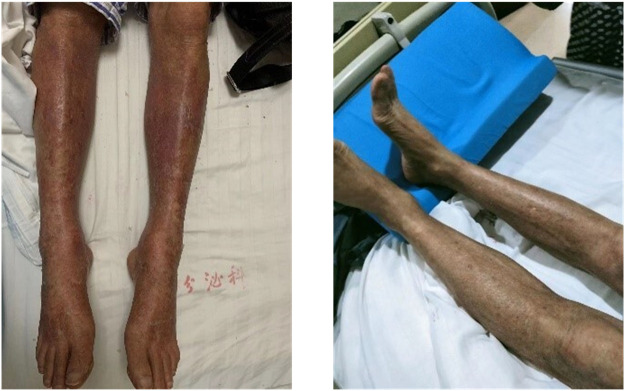
Clinical features of the patient. **(A)** Rashes on the leg on admission; **(B)** Rashes on the leg post-recovery.

The patient was a 73-year-old male who developed a systemic skin rash after taking metronidazole and other proprietary Chinese medicine a couple of months before admission. He had done nothing about the rash before he developed icteric sclera. He was hospitalized for further treatment. The patient had a history of chronic eczema and was allergic to moxifloxacin; he denied a history of diabetes. The patient did not drink alcohol and had quit smoking for the last 30 years. No diabetes was reported in his family. He was generally in a good condition at admission, sane, and conscious. Physical examination showed icteric sclera and dark red flaky rashes all over the body (Figure 1), however, no other obvious abnormality was detected. Lab tests showed elevated levels of liver enzymes [aspartate transaminase 150 U/L (reference range, 9–50 U/L) and alanine aminotransferase 81 U/L (reference range, 15–40 U/L)]. Total bilirubin increased to 100 umol/L (reference range, 2–20.4 umol/L) along with high levels of direct bilirubin 78 umol/L (reference range, 0.2–7 umol/L) and indirect bilirubin 22 umol/L (reference range, 0.9–13.7 umol/L). Drug dermatitis was diagnosed after a skin biopsy. He underwent liver protection therapy daily and was administered methylprednisolone. Blood glucose levels were monitored daily and showed within the normal range (fasting blood glucose 4.0– 6.0 mmol/L, postprandial blood glucose 4.4–7.8 mmol/L). On the 9th day of hospitalization, the patient had hyperglycemia with a sudden increase in blood glucose level to 42 mmol/L and was positive for the presence of urine ketones. Values of arterial pH, bicarbonate, and base excess were normal. After considering the diagnosis of diabetic ketosis, he was treated with IV low-dose insulin plus saline. Given the effects of steroids on blood glucose, loratadine and cetirizine were used to treat the skin rashes instead of intravenous steroid administration. Insulin was subcutaneously injected after the urine ketone test was negative. To investigate the cause underlying the sudden hyperglycemia, further laboratory tests were conducted. The glycated hemoglobin (HbA1c) value was 7% and no diabetic complications were found. Fasting C-peptide showed a very low level, 0.11–0.13 ug/L (reference range for fasting c-peptide, 1.10–4.40ug/L). The patient tested negative for islet-related antibodies (glutamic acid decarboxylase antibody and anti-insulin antibody) and his pancreatic enzymes [amylase 399 U/L (reference range, 35–135 U/L) and lipase 153 U/L (reference range, 0–60 U/L)] were elevated. Tests for other autoimmune-related antibodies were also negative. A case of pancreatitis was suspected. However, there were no obvious signs of pancreatitis in the abdominal magnetic resonance imaging (MRI) scans or abnormality as evidenced by the results of the magnetic resonance cholangiopancreatography. Finally, the patient was diagnosed with FT1DM. Considering FT1DM is often related to viral infections, tests associated with viral infections were conducted. The antibodies for hepatitis virus and several serological tests for other viruses (parvovirus, cytomegalovirus, rubella virus, adenovirus, respiratory syncytial virus, influenza virus and parainfluenza virus) were all negative. However, the titer of EBV-DNA (4.744*10^4copies/ml [reference range, <1*10^3copies/ml]) in lymphocytes was significantly high. The percentage of the T8 cells (CD45^+^CD3^+^CD8^+^) was 1.5-fold the normal range (normal range 16.40–33.76%), which implied the enhanced activation of CD8 T lymphocytes, while that of the NKT cells and T4 cells (CD45^+^CD3^+^CD4^+^) and T4/T8 decreased, suggesting suppressed immunity (Table 1).

**TABLE 1:** Immune monitoring AND HLA-DNA typing.

Immune monitoring			HLA-DNA typing			
Types of immune cells	Results	Normal range	Allele	HLA-A	HLA-DRB1	HLA-DQB1
T4cellsCD45+CD3^+^CD4^+^	0.71*10^9/L	0.20–1.82*10^9/L	Allele1	A*02:07	DRB1*12:02	DQB1*03:01
T8cellsCD45+CD3^+^CD8^+^	1.63*10^9/L	0.13–1.35*10^9/L	Allele2	A*11:01	DRB1*12:02	DQB1*03:01
T4/T8	0.44	0.89–2.01				
T4cells%CD45+CD3^+^CD4^+^	24.15%	24.93–45.57%				
T8cells%CD45+CD3^+^CD8^+^	55.28%	16.4–33.75%				
NKTcells% CD45^+^CD3^+^CD56^+^	0.53%	3.00–8.00%				

**FIGURE 2:**
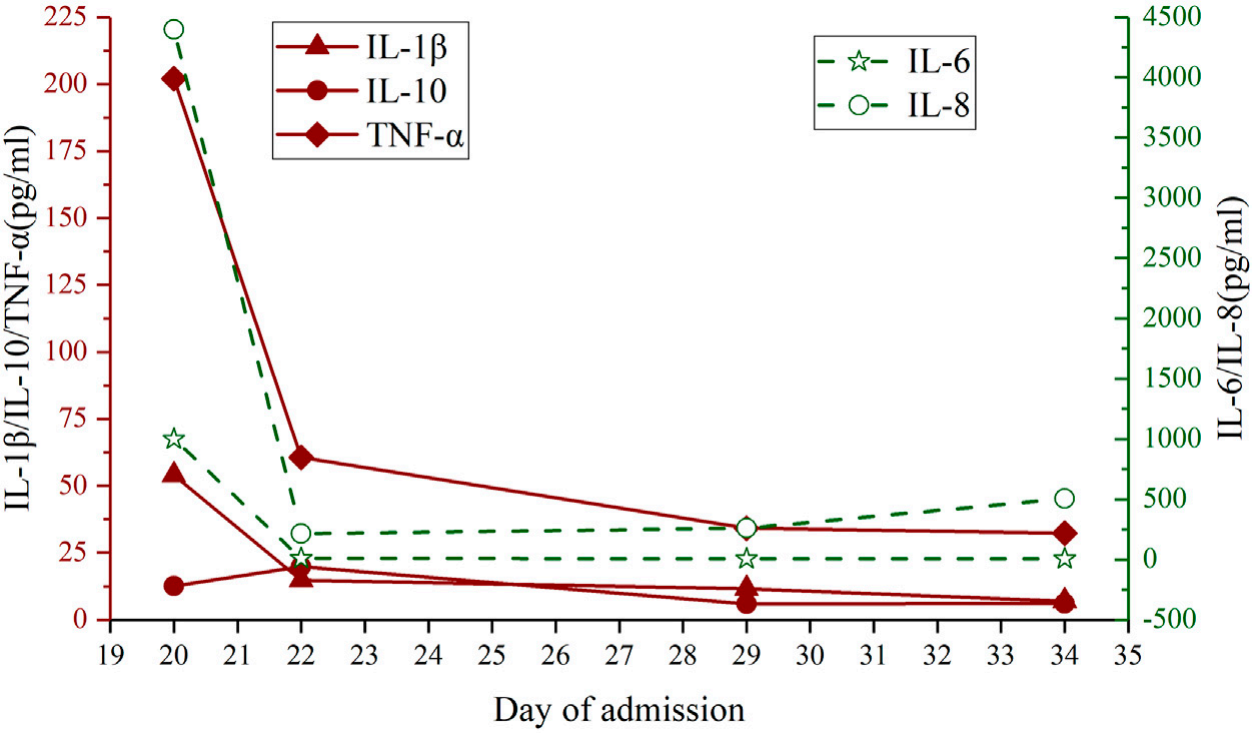
Changes in IL-1β, IL-6, IL-8, IL-10, and TNF-α levels through the clinical course.

His blood glucose levels and skin rash conditions were relatively stable until the 18th-day post-hospitalization, whereby he underwent an abdominal enhanced computed tomography (CT) examination for further pancreatic evaluation, post which, he developed severe fever and chills, with his temperature fluctuating between 38.5 and 39.5°C. Blood pressure was in the 80–90/40–50mmHg range. Laboratory examinations suggested an increased count of leukocytes [9.54 × 10^9/L (reference range, 3.5–9.5 10^9/L)] and eosinophils [35.1% (reference range, 0.4–8%)]. CRP and procalcitonin were also elevated. Blood culture and bacteriological tests were negative. Cytokines [IL-6 > 1000pg/ml (normal range 0.00–5.90pg/ml), IL-8 4399.00pg/ml (normal range 0.00–62.00pg/ml)] increased significantly (Figure 2). The pathological morphology of blood cells showed an 8% heterotypic lymphocyte population. Chest CT scan suggested some inflammation in the lower lobes of both lungs. The abdominal CT scan showed a small peritoneal effusion, however, there were no signs of pancreatitis. Systematic methylprednisolone (60mg, ivgtt qd) was administered. Imipenem was used for preventing bacterial infection. The patient’s general condition was gradually stabilized towards the 32nd-day post-admission. Unfortunately, when the administration of IV methylprednisolone was converted to oral prednisone and thymic pentapeptide to improve the patient’s low immunity, he developed anaphylaxis, and the rash became significantly worse than before. The percentage of eosinophils was now 31.6% (reference range, 0.4–8%), the titer of the EBV-DNA [1.28*10^5copies/ml (reference range, <1*10^3copies/ml)] in lymphocytes was higher than before, while the serum C-peptide levels were below the detectable limit (c-peptide < 0.01 ug/L), and the islet-related antibodies were still negative. Allergy to prednisone and thymic pentapeptide was suspected and immunoglobulin, antihistamine drugs, along with methylprednisolone, were systemically intravenously administered. Intensive insulin treatment was continued. The levels of blood glucose, liver damage, and general condition of the patient were relatively stabilized and oral dexamethasone was administered. The retest of immune cells showed no obvious abnormalities. Finally, he was discharged on the 38th-day post-hospitalization. Post-discharge, the patient continued insulin therapy and dexamethasone which was gradually tapered and eventually discontinued after one month. In the second month of the follow-up, the patient was stable. His rash improved significantly (Figure 1). We tested for genes associated with diabetes, however, no hyperglycemia-related pathogenic gene mutations were found by whole-exome analysis. He did not have a reported human leukocyte antigen (HLA) type (Table 1) associated with FT1DM. After 6 months of follow-up, the patient still requires regular subcutaneous insulin injections and his blood glucose levels are well within control.

## Discussion

In this case, the blood glucose levels increased suddenly and rapidly progressed to ketosis with the HbA1c level at 7%. Fasting serum C-peptide levels were 0.11 ng/ml and undetectable later. In addition, the patient tested negative for islet-related antibodies. There was a slight elevation in the levels of pancreatic enzymes but no obvious pancreatitis was observed in both MRI and CT scanning. All the above abnormalities were in accordance with the diagnostic criteria of The Committee of the Japan Diabetes Society, FT1DM was diagnosed (Imagawa et al., 2012). High titers of EBV-DNA in lymphocytes were detected, while there was no significant increase in antibody titers against other viruses. The patient’s drug-induced dermatitis was repeatedly aggravated, the EBV-DNA titer increased continuously, and the reexamination of serum C-peptide level was lower than the detection limit. When the patient was admitted, icteric sclera was observed and the results of the blood examination suggested elevated bilirubin levels. After admission, the patient had repeated fever, activation of CD8 T lymphocytes, the appearance of atypical lymphocytes, and a cytokine storm, which could not be controlled by antibiotic administration. These were consistent with the manifestations of an EBV infection. His condition worsened after administering some drugs even prednisone (iodinated contrast agents, prednisone, and thymic pentapeptide). Although prednisone is widely used as an antiallergic drug, there are still some reports of hypersensitivity to prednisone (Nucera et al., 2002; Kim and Kim, 2014). Corticosteroid itself, or the additives and vehicles in corticosteroid preparations may induce hypersensitivity reactions (Otani and Banerji, 2016). However, it is hard to differentiate whether prednisone or the additive or pentapeptide that induced the patient’s hypersensitivity in this case. But we did see that his dermatitis got worse after using those above drugs. Finally, FT1DM was diagnosed, and we think that drug hypersensitivity and EBV infection together led to FT1DM in this case (Supplementary Figure S1).

FT1DM caused by DIHS has been reported previously. DIHS is a severe adverse drug reaction, often involving multiple internal organs and autoimmune sequelae, including thyroid diseases, diabetes mellitus, and systemic lupus erythematosus. It is characterized by fever, rash, hepatitis, lymphadenopathy, and eosinophilia. Currently, DIHS is thought to be caused by a combination of drug-specific and virus-specific responses (Miyagawa and Asada, 2021). DIHS is often associated with carbamazepine, phenytoin, phenobarbital, Zonisamide, methylil, dapsone, sulfasyridine salazo, and allopurinol, while DIHS due to other drugs is relatively rare. Previous studies show that DIHS is often associated with HHV6 and reactivation of other herpesviruses (human herpesvirus 7, EBV, and cytomegalovirus) (Seishima et al., 2006; Shiohara and Kano, 2007). The question therefore was, is this a case of FT1DM caused by reactivation of viral infection induced by DIHS? According to the diagnostic criteria for DIHS proposed by the Japanese panel, DIHS is a delayed hypersensitivity reaction, usually manifesting as a rash 3 weeks after the administration of the drug and is often characterized by abnormal liver function with an elevated level of alanine aminotransferase (Shiohara et al., 2007). However, the patient developed a systemic rash only after taking the drug and had a slight abnormality in levels of liver enzymes. Thus, this case did not meet the above diagnostic criteria and we didn’t consider this as a case of FT1DM caused by DIHS. Considering the relationship between HHV-6, DIHS and FT1DM, testing for HHV-6 should be considered. However, HHV-6 infection replicates primarily in CD4(+) T lymphocytes, which is different from our case (Takemoto et al., 2009).

We have good reason to think that the drug allergy and EBV infection played a synergistic role in the development of FT1DM in this case. Viral infection can promote the development of drug-related rashes, such as an increased risk of skin eruptions in patients with EBV infection whilst using beta-lactam antibiotics (Chovel-Sella et al., 2013). DIHS is associated with infection of the virus and the viral infection itself can cause a rash (Mims, 1966). Notably, there has been a case report, wherein ampicillin caused the reactivation of EBV, resulting in cutaneous eruption (Saito-Katsuragi et al., 2005). Therefore, the relationship between rash, drug allergy, and virus infection is tight. In this case, drug hypersensitivity was accompanied by the infection of EBV, thereby leading to the occurrence and progression of FT1DM.

The occurrence of FT1DM is also related to genetic factors. According to recent studies, the class II HLA genotype is associated with the onset of FT1DM (Imagawa et al., 2008). Previous studies suggest that HLA-DR4-DQ4 is more frequent in FT1DM and the *CTLA4* gene contributes to an enhanced susceptibility for FT1DM (Imagawa et al., 2005; Kawasaki et al., 2008). Further, studies show that DRB1*04:05-DQB1*04:01 increases the risk of FT1DM (Kawabata et al., 2009). Another study also reports similar results; the DRB1*09:01‐DQB1*03:03 haplotype is positively associated with FT1DM, while the DRB1*01:01-DQB1*05:01, DRB1*08:03-DQB1*06:01, and DRB1*15:02-DQB1*06:01 haplotypes are negatively associated with FT1DM (Tsutsumi et al., 2012). Although DRB1*1501-DQB1*0602 is negatively related to T1DM and exerts a protective effect, the haplotypes that are negatively associated with FT1DM do not show any protective effects (Kawabata et al., 2009; Tsutsumi et al., 2012). However, the HLA typing in this patient showed frequencies that are relatively common in the general population, and no reported cases of FT1DM significantly correlated with these genotypes were found. Additionally, no hyperglycemia-related pathogenic genes were found by the whole-exome analysis. Therefore, genotype maybe not be an essential factor for FT1DM.

The use of steroids in the treatment of drug allergic dermatitis exerts definite effects, however, there is also the risk of hyperglycemia. A study reported that 70% of patients had elevated blood glucose when treated with glucocorticoids in hospitals; this was related to the time of glucocorticoid use (Fong and Cheung, 2013). Glucocorticoids can promote lipolysis in adipose tissues, hepatic gluconeogenesis, increase circulating glucose concentration, and reduce peripheral insulin sensitivity. In this case, the patient’s insulin level is very low and therefore blood glucose levels were elevated mainly due to an absolute lack of insulin. However, use of steroid may have aggravated the hyperglycemia. And the patient only had slightly elevated levels of pancreatic enzymes. Thus, the possibility of FT1DM caused by acute pancreatitis was also low.

Therefore, we considered this as a case of FT1DM caused by drug allergy and EBV infection. Thus far, there has been one case report of FT1DM in multiple myeloma patients complicated due to EBV infection (Fujiya et al., 2010), but there has been no report on FT1DM caused by drug hypersensitivity and EBV infection. FT1DM is dangerous and progresses rapidly. FT1DM should be therefore considered in patients with severe hyperglycemia showing a rapid progression to ketosis without high HbA1c levels; timely treatment should be appropriately given. FT1DM occurs in a certain proportion of the Asian population. It is known that FT1DM is closely related to viral infection. However, many questions on the etiology of FT1DM need further investigation.

## Conclusion

Herein, we report the first case of FT1DM due to drug hypersensitivity and EBV infection. Through this rare case report, we emphasize the importance of the relationship between drug hypersensitivity, EBV infection and FT1DM, and vigilance for the occurrence of FT1DM among hypersensitive individuals. Drug hypersensitivity and EBV infection are not rare in the population. The drug hypersensitization in hypersensitive individuals and the infection of virus needs vigilance. FT1DM is a life-threatening disease. Early identification and treatment are of great help to improve the prognoses of these patients.

## Data Availability

The original contributions presented in the study are included in the article/Supplementary Material, further inquiries can be directed to the corresponding author.

## Supplementary Material

Supplementary Figure S1 Causes of FT1DM in the case.

## Abbreviations

FT1DM, fulminant type 1 diabetes mellitus; T1DM, type 1 diabetes mellitus; DIHS, drug-induced hypersensitivity syndrome.
