# Supplementary figures and images for: Fulminant Type 1 Diabetes Mellitus Associated With Drug Hypersensitivity and Epstein–Barr Virus Infection: A Case Report
Source: Front Pharmacol. 2022 Jul 8;13:884878. doi: 10.3389/fphar.2022.884878 (PMC9305474; doi:10.3389/fphar.2022.884878)

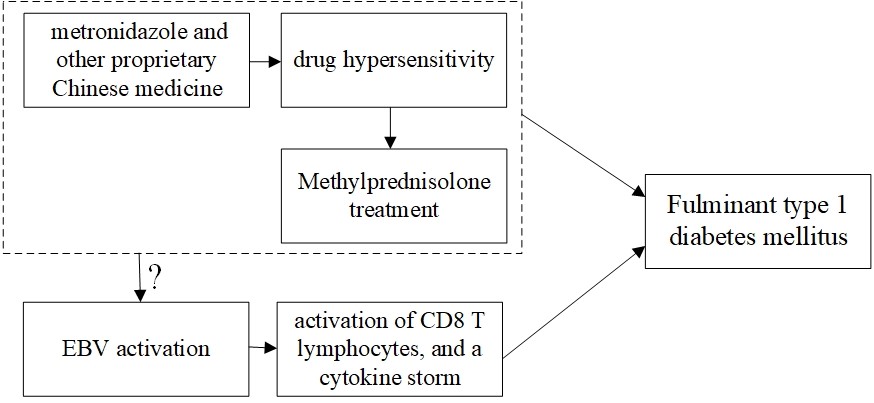

Supplement: Supplementary file 1 [file Image1.JPEG]
